# Unite the Forces

**Published:** 1898-12

**Authors:** George H. Chance

**Affiliations:** Portland, Ore.


### UNITE THE FORCES.[1]

BY GEORGE H. CHANCE, D.D.S., M.D., PORTLAND, ORE.

When President McKinley issued his proclamation calling for volunteers to drive the Spaniards from Cuba, the latent fighting forces, which were scattered far and wide, had to be brought together, organized, and properly drilled and equipped before they could become effective and utilized. Not only so, there had to be a union of sentiment in the purpose for which the fighting bodies were organized to bring about the splendid achievements of our army and navy, which have resulted in such glorious victories to our arms, making a proud epoch in the onward march of our nation, in the emancipation of millions of human beings from the tyranny of a grovelling superstition united to a despotic power.

It is sometimes said by the unthinking that there is no sentiment in business, which is certainly a mistake, for had there been no national sentiment in behalf of the starving Cubans and no

---

[1] Read before the Pacific Coast Dental Congress, August 24, 1898.

indignation aroused at the blowing up of the "Maine," this country would not have voted the men and money it has done to prosecute the war against Spain, and which in the short period of less than five months has terminated so successfully in behalf of suffering humanity, and of placing this nation in a position to command the respect, if not the admiration and love, of the whole civilized world.

Now, this matter of sentiment, that sometimes is thought too lightly of, when properly directed and united for a fixed purpose, becomes a great moral force, underlying all true success in life, whether individually or collectively, and, think as we may, it is this principle which brings us together as members of the Pacific Coast Dental Congress, imbued, I trust, with a common sentiment united for a fixed purpose, that each may do his part in making its sessions a new point of departure, in lifting our profession, on the Pacific Coast at least, to a higher plane of moral and professional excellence, and this will be done if a well-directed sentiment is allowed to govern our actions. This paper has not been written so much for the purpose of emphasizing the value of this sentiment among the ethical members of the dental profession, however valuable that may be, but is an effort to try to arouse a sentiment which shall be the instrument to unite the forces which go to make up the medical and dental professions, for we cannot deny the facts, if we would, that there is not that union of sentiment and fellowship between these two bodies which it would seem ought to exist. Not that there is any special antagonism between them, but that in a sense, as professional men, they are strangers to each other, quite as much so as are lawyers and physicians; useful to each other at times, but ordinarily seeming to have no special interest in common. This to the thinking ethical dentist is all wrong, and ought not to be.

Let us trace the cause and perhaps we may find a remedy for the complaint, and while we say all hail to the grand old profession of medicine, yet your essayist must be true to history, which teaches that, in the not very distant past, medicine, neglecting one of its functions, permitted the dental branch to be broken off from the parent stock, to be mutilated and kicked about by every tinker or blacksmith who happened to come along, until at last a few far-seeing medical men, of an appreciative turn of mind, picked up the apparently lifeless branch and, lopping off the dead boughs, planted it in a new but nevertheless medical soil, and by careful and persistent cultivation on the part of the successors of the original planters, from time to time adding such compost to the soil as the case seemed to require, there has been produced from that broken branch a large, healthy tree of thrifty growth, bearing fruit of a quality not to be excelled by the old medical tree itself. Of course, the young tree, like the old, has to be sprayed from time to time in order that the parasites shall do as little damage to the fruit as possible.

Now, if this dental tree is of the same stock as all other branches of the medical tree, though of an independent growth and development, it logically follows that there should be a full and free exchange of the fruits of each without involving arbitrary conditions by which such exchange may be made; therefore, whatever medical men of the past may once have thought of dentistry, the stomatology of to-day, as taught and practised, is no longer an unknown quantity, but is truly as much a branch of the healing art and entitled to as full recognition, as such, as are the other branches termed specialties in medicine.

It is often urged by medical men, when spoken to on the subject, that the dental graduate, to be in touch with the medical profession, must possess the medical degree; but why should this be? The medical colleges have not now nor ever have had the facilities or the inclination to teach students in dentistry, and perhaps it is well that it is so; but because this branch of medicine is of independent growth, is no good reason why physicians and dentists should not be in full professional touch with each other.

It was of vital importance to this nation that General Shafter should be in full touch with Rear-Admiral Sampson at the taking of Santiago; notwithstanding, Shafter and Sampson were not educated in the same school of war; but we must not forget that in each of the schools in which these men received their fighting education, the line of studies up to a given point were the same, the fundamentals in both schools being identical, so that in their conferences and consultations they thoroughly understood each other, and were thus enabled to act in concert for the common interest. Now, why should not the same sentiment exist between the graduate in medicine and the graduate in dentistry in waging war against human disease? It is equally true in dental and medical schools, as in the schools referred to, that up to a given point the line of studies are the same. All the fundamentals in both schools being identical, and as thoroughly taught in the one as in the other, thanks to the scientific progress made in all departments of human knowledge, they are united in the preliminary preparation on the part of the students. The unnatural estrangement which now separates the two professions will soon be a thing of the past, and in its place a feeling of professional brotherhood will be aroused which does not at present exist. In order to bring this about there should not be a disposition to flatter the physician on the part of the dentist, nor on the part of the physician to flatter the dentist, for the one is the peer of the other, and though the professional service rendered may differ in character, it does not in quality. Let us then as dentists do all we can in a dignified professional way to bring ourselves into closer touch with the members of the mother profession, and this we may do by always applauding the action of the medical profession in the establishment, which it did a few years ago, of a stomatological section as part of all international medical congresses. It will then only remain for the State medical societies throughout the Union to follow the example of the International Congress, to bring about a greater respect for the members of each profession, and this union, when accomplished, will teach us how much each has to learn before one is competent to sit in judgment over his professional brother, and to hasten forward the time of this much-wished-for professional millennium.

It is suggested, if not thought to be too presumptive on the part of a dentist to make it, that the local medical societies throughout the land amend their by-laws so as to admit as associate members all graduates of reputable dental colleges, they paying like dues and having like privileges with the other members, except the right to vote on the business affairs of the society, and let the same thing be done, on the same terms, in all local dental societies towards medical men, giving them all the rights and privileges of the society except the right to vote in governmental affairs.

It is further respectfully suggested to our medical brethren that they use their influence to have the medical colleges establish, in addition to the special chairs already established, chairs on dental pathology, that the diseases of the dental organs and associate parts, often so closely related to the eye, ear, and other reflex disturbances of the body, may be better and more generally understood than at present by students in medicine.

Then all would be better able to discriminate between the ethical and the non-ethical in both professions, and thus, I take it, we would unite all the reputable forces of the medical and dental professions in the legitimate war each is trying to wage, more or less successfully, against human disease.

